# Everyday struggles to stay healthy: experiences of people at high risk of stroke residing in low socioeconomic urban neighbourhoods in Stockholm

**DOI:** 10.1186/s12889-026-29974-7

**Published:** 2026-10-16

**Authors:** Lisette Farias, Lisa Holmlund, Omar Hawajri, Susanne Guidetti, Ann-Helen Patomella

**Affiliations:** 1 Division of Occupational Therapy, Department of Neurobiology, Care Sciences and Society, Karolinska Institutet, Stockholm, Sweden; 2 Division of Nursing, Department of Neurobiology, Care Sciences and Society, Karolinska Institutet, Stockholm, Sweden; 3 Department of Women’s Health and Allied Health Professionals, Karolinska University Hospital, Stockholm, Sweden; 4 Academic Primary Health Care Centre, Region Stockholm, Stockholm, Sweden

**Keywords:** Stroke prevention, Everyday life, Underserved populations, Health inequalities, Health promotion, Primary health care

## Abstract

**Background:**

Residents in low socioeconomic urban neighbourhoods are disproportionately exposed to modifiable risk factors, yet their engagement in health-promoting behaviours remains poorly understood from a lived experience perspective. This study aims to explore how residents at high risk of stroke experience and engage in everyday health-promoting activities within their neighbourhoods.

**Methods:**

A qualitative study was conducted using semi-structured interviews with eighteen adults residing in low socioeconomic urban neighbourhoods in Sweden who were identified as being at high risk of stroke. Data were analysed using reflexive thematic analysis, following Braun and Clarke’s approach, to capture participants’ experiences at multiple levels.

**Results:**

Three themes were developed: When caring for others becomes a barrier to one’s own health, When economic conditions hinder engagement in health-promoting behaviours, and When health-promoting spaces become stressful and unsafe. Participants described how caregiving responsibilities, financial constraints, and stress limited their capacity to engage in health-promoting activities. Although participants often had knowledge of healthy behaviours and expressed motivation, their efforts were disrupted by competing demands and constrained life conditions. Spaces typically framed as supportive of health, such as gyms, were not always experienced as accessible and could instead evoke discomfort, stress, or insecurity. Social support was present but often insufficient to overcome these cumulative challenges.

**Conclusions:**

Engagement in health-promoting activities among individuals in low socioeconomic urban contexts is shaped by the intersection of relational, structural, and contextual factors, rather than by individual motivation alone. Health promotion may be experienced as an additional burden within already constrained lives. Effective health promotion strategies should therefore move beyond individual behaviour change and adopt context-sensitive, multi-level approaches that are integrated into everyday life by fostering meaningful and varied forms of engagement. Such approaches are essential to reach and ensure the active participation of residents of low socioeconomic areas in health-promoting initiatives.

**Clinical Trial registration:**

Not applicable.

## Background

Social inequalities in stroke incidence and mortality are a persistent public health concern, both globally and within high-income welfare states such as Sweden [1, 2]. Recent global analyses show that, despite advances in prevention and acute care, substantial socioeconomic disparities continue to shape stroke risk, incidence, and survival [3]. Socioeconomic status (SES), commonly operationalised through education, income, and occupation, is therefore well-established as a determinant of stroke incidence, severity, and stroke-related mortality [1]. Nationwide studies from Sweden illustrate these inequalities, showing that individuals with low SES, especially those with the lowest income, had an increased risk of death within 3-months after a stroke [4] and that living in a low SES neighbourhood, characterized by unemployment, low income and education levels, and receipt of social welfare, increases the risk of ischemic stroke before the age of 50 by 12–16% [5].

Research has demonstrated that social gradients in modifiable risk factors contribute substantially to inequalities in stroke burden [6]. Hypertension, smoking, obesity, and harmful alcohol use are consistently more prevalent among individuals with lower SES, contributing to an increased risk of stroke across their life course [1, 7]. These inequalities extend beyond stroke incidence since individuals with lower SES also tend to experience stroke at a younger age and with greater severity, followed by poorer functional outcomes and worse overall health and quality of life [8–10]. Socioeconomic disadvantage also influences care-seeking behaviour [2], as individuals with lower SES are less likely to recognise stroke warning signs or to be aware of modifiable risk factors, increasing the likelihood of delayed care-seeking [11] and receiving inadequate health care [8, 9]. These socioeconomic gradients persist beyond the acute phase, as persons living in low SES areas after a stroke report greater reductions in participation in meaningful leisure activities [12], thereby reinforcing long-term health inequities.

Although stroke incidence in Sweden has declined at the population level, this reduction has been uneven, with slower progress observed among socioeconomically disadvantaged populations [13, 14]. In response, national and regional initiatives have aimed to reduce inequalities in stroke prevention and care. For instance, the Swedish Stroke Register (Riksstroke), a validated national quality register, is central to monitoring equal care and improving stroke services. It covers all 72 Swedish hospitals that receive acute stroke patients and includes follow-up at 3 and 12 months [15–17]. Additionally, municipality-based prevention initiatives, including programs promoting a physically active lifestyle, have been implemented in selected areas [18]. Despite these efforts, prevention initiatives in Sweden have shown limited success in reaching socioeconomically disadvantaged populations, often due to a lack of recruitment strategies and community involvement [19, 20].

Despite the well-documented socioeconomic inequalities in stroke incidence and outcomes worldwide, research exploring people’s lived experiences to inform more equitable prevention remains limited [21, 22]. In particular, studies on lifestyle changes and health-promoting activities, such as physical activity, among low SES populations are scarce [23, 24]. Moreover, contextual factors that affect low SES groups’ engagement in health-promoting activities, such as environmental constraints, social norms, and competing life demands, are often underreported, limiting the development of interventions tailored to specific settings [25]. Qualitative inquiry is therefore essential for illuminating the lived experiences of individuals at risk of stroke and for understanding the contextual constraints and sociocultural dynamics that influence engagement in health-promoting activities in low socioeconomic settings. Such insights are necessary to inform health promotion approaches that extend beyond individual behaviour change and address contextual barriers [23]. Without such understanding, individuals at the highest risk of stroke are likely to remain the least able to participate in preventive efforts, thereby perpetuating existing health inequities [21].

This study forms part of the initial exploratory phase of developing a contextually tailored stroke prevention intervention in low SES urban neighbourhoods in Sweden. The aim of this study was to explore how residents at high risk of stroke experience and engage in everyday health-promoting activities within their neighbourhoods.

## Methods

### Research design

This study employed an exploratory qualitative design informed by social constructionism to capture the complexity of residents’ lived experiences of engagement in low SES neighbourhoods. This approach enabled an exploration of participants’ subjective experiences and the social contexts that shape their sense-making, consistent with a social constructionist orientation [26]. This orientation supported an understanding of how participants’ meanings, perceived expectations, and struggles were situated within their socio-cultural and contextual environments [27]. The study is reported in accordance with the Reflexive Thematic Analysis Reporting Guidelines (RTARG) [28].

### Positionality

All the authors work at a Swedish university and have research experience in health sciences. LF and LH have extensive training in conducting qualitative research with participants from different cultural and socioeconomic backgrounds. OH is a male nurse with experience in health practice in nursing care and public health, and has worked as a research assistant on various projects. SG and A-H P are female researchers with extensive experience in developing, evaluating and implementing stroke prevention programs and complex interventions within health sciences. LF and OH are non-Swedish speakers, while SG, LH, and A-H P are native Swedish speakers, contributing a range of perspectives to the research process.

### Setting

The two selected low SES urban neighbourhoods were situated in different parts of Stockholm, one in the northern and the other in the southern area of the city. Both are characterised by a complex socioeconomic context in which challenges coexist with ongoing investments. According to a municipal survey of these neighbourhoods conducted in 2024, they have the highest unemployment rate of any area in Stockholm. The neighbourhoods are also characterised by a high level of cultural diversity, with approximately 75% of residents having a foreign background, defined as either being born abroad or having two foreign-born parents. They are also associated with open drug scenes and criminal networks, making them similar to other vulnerable areas in Stockholm. These neighbourhoods often have a local centre with services such as primary healthcare centres, a library, schools, and good communications.

Regarding health promotion and stroke prevention in Sweden, primary healthcare is responsible for both identifying and long-term managing modifiable risk factors. Primary healthcare is largely financed by regional and municipal income taxes. However, adherence to national stroke-prevention guidelines varies considerably across primary healthcare centres [29]. In low-socioeconomic urban areas, stroke prevention is affected by structural and social barriers, including language barriers, limited health literacy, reduced trust in institutions, and challenges navigating the healthcare system, which increase the likelihood of refraining from seeking healthcare despite perceived need [30].

### Recruitment and participants

Recruitment was conducted by LF, OH, and A-H P in August to December, 2025 and included several strategies tailored to reach socioeconomically disadvantaged groups. The team presented the study at several local venues, e.g., an adult education centre offering free Swedish language courses, and at Food and Christmas festivals organised by the municipality. At these venues, the research team distributed study information verbally and via flyers, inviting potential participants to have their blood pressure measured, weigh themselves, and complete the Stroke Risk Assessment, a stroke risk screening tool developed by the American Stroke Association [31]. These assessments were used to identify individuals who met the inclusion criteria of high stroke risk and included elevated blood pressure (> 140/90 mmHg, which is in accordance with Swedish recommendations) or diagnosed hypertension with at least two modifiable stroke risk factors (e.g., smoking, diabetes, low physical activity, or overweight). Exclusion criteria included insufficient proficiency in Swedish or English to participate in an interview and residence in a care facility.

After completing the Stroke Risk Assessment, participants who met the criteria were asked to indicate their interest in being contacted by the research team and were provided with the research team’s contact information. Follow-up communication occurred via email or telephone to schedule interviews at times and locations convenient for participants. By inviting all interested individuals who met eligibility criteria, the study aimed to minimise selection bias and avoid unintentionally favouring or excluding any subgroup of potential participants [32].

A total of 18 participants agreed to take part in an interview. The mean age of the participants was 54.5 years (SD = 6.12), and the majority were women (*n* = 11). Fourteen participants reported being married or living with a partner, while two were single and two were divorced. Most participants had children (*n* = 15), and the mean years of education was 12.5 years (SD = 3.01). Participants were primarily from Sweden (*n* = 4), Asia (*n* = 7), Africa (*n* = 6), and South America (*n* = 1). The mean monthly household income (the sum of everyone’s income living in the same home) after taxes was 40,746 SEK (SD = 20,223). The average income in Sweden for the age range of our participants, 45–64 years, is 47,000 SEK, and after taxes, approximately 33,000 SEK per person [33]. Additionally, half of the participants reported that they were able to manage their household expenses.

### Data Collection

LF and OH conducted joint interviews with 10 participants, OH and A-H P conducted joint interviews with 2 participants, and OH interviewed 6 participants. The interviews were conducted using a semi-structured interview guide developed by the research team for this project and a series of related studies. A detailed description of the interview guide has been published elsewhere [34]. Interviews were chosen in this exploratory phase because they offer flexibility to follow up to gain a clearer understanding of participants’ experiences, ask probing questions, and adapt to participants’ responses [35]. The interview guide consisted of two parts: background information questions and questions about participants’ lifestyle and participation in health-promoting activities. It included 12 open-ended questions, with space for follow-up questions to clarify and promote participants’ further discussion and elaboration. The interviews included questions about experiences of previous participation in health-promoting activities in the local neighbourhood, challenges and opportunities to participate in current or new health-promoting activities and to initiate a lifestyle change, and perceived support for starting a lifestyle change.

The interviews were conducted face-to-face at locations chosen by the participants, such as a community centre (offering free Swedish-language courses), a library, a shopping mall, and the homes or workplaces of a few participants. The research team offered the option to conduct interviews in English or Swedish; all participants chose Swedish, with varying levels of language proficiency. To address variations in language proficiency, the interviewers adapted their interviewing style by using simple and clear language, avoiding jargon, repeating or rephrasing questions, providing examples, allowing additional time for responses, and creating opportunities for clarification. All interviews, which ranged from 28 to 51 min, were audio-recorded and transcribed by OH.

### Ethical considerations

The study was conducted in accordance with the principles of the Declaration of Helsinki [36]. Ethical approval was obtained from the Regional Ethical Review Board in Stockholm (Dnr. 2024-05142-01). All participants were informed about the study’s aim and procedures, as well as their right to withdraw at any time without consequences, both orally and in writing. Written informed consent was obtained from all participants prior to data collection. As a token of appreciation for their time, participants received a movie ticket. All collected data were handled confidentially and stored securely in accordance with data protection regulations.

### Data analysis

Data were analysed using Braun and Clarke’s [37] six-phase approach to reflexive thematic analysis, following an inductive, iterative process that involved movement back and forth between phases. Reflexivity and analytic depth were enhanced through the research team’s involvement in recruitment activities in the selected low SES neighbourhoods, which enabled them to gain firsthand insight into the physical environments, social dynamics, and everyday contexts described in the interviews. These experiential understandings were continually revisited and critically examined in relation to the dataset, thereby informing ongoing team discussions about the contextual nuances that shape participants’ accounts. As recommended by the Reflexive Thematic Analysis Reporting Guidelines (RTARG) [28], this experiential knowledge was critically reflected upon to consider how it informed theme development and the meaning-making process.

All transcripts were systematically coded by LF using Atlas.ti 25, with equal analytic attention to each data item. The software supported a transparent and organised coding process, enabling efficient navigation of the dataset and facilitating an accessible review of codes. Following initial coding, the coded dataset was grouped into preliminary themes by clustering codes that shared underlying meanings or conceptual patterns across participants’ accounts. Meanings were sought to illuminate how broader social, cultural, and material conditions shaped participants’ experiences of health and everyday life. These preliminary themes were then reviewed against the coded extracts and the full dataset to refine coherence and analytic fit. Several rounds of mapping in Atlas.ti 25 facilitated a flexible reorganisation of codes and themes, supporting the analytic creativity that RTARG [28] recommends. Through ongoing discussions, themes were further refined to capture each theme’s central organising concept, as advised in RTARG [28]. This process was led by LF and supported by A-H P, and was finalised when the rest of the team revised whether the themes and their names captured their central organising meanings. The analysis was conducted in Swedish, a language managed by the research team, and after finalising the process of writing the results, the quotes were translated into English and revised by the team.

To ensure trustworthiness, the study’s analytic process was evaluated using Braun and Clarke’s [27] guiding questions for assessing quality in reflexive thematic analysis. These questions supported consideration of the rationale for choosing reflexive thematic analysis, clarity regarding the reflexive approach employed, and alignment between the study’s epistemological positioning, data collection methods, and analytic procedures.

## Results

**Fig. 1:**
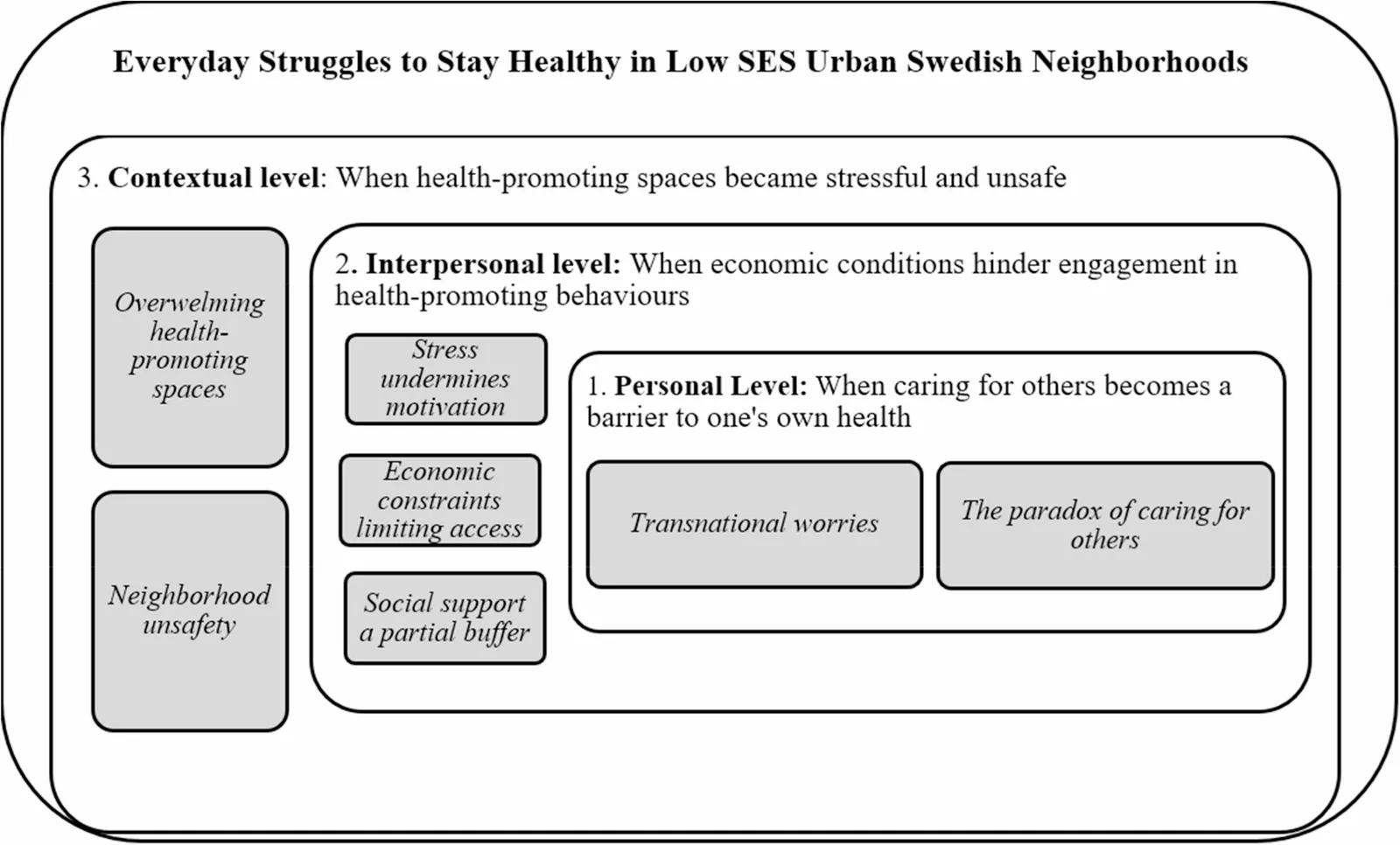
Overarching theme, themes, and subthemes

An overarching theme, three themes, and subthemes were developed through reflexive thematic analysis (Fig. 1). The overarching theme ‘Everyday struggles to stay healthy’ illustrates that staying healthy in a low SES urban context is not merely influenced by isolated barriers but is shaped by ongoing processes of negotiation among personal demands, structural/economic constraints, and (un)safe environments. These negotiations are navigated at a multi- and interdependent system (personal, interpersonal, and contextual levels) that systematically produces, redirects, and sometimes undermines efforts to maintain health. These layered conditions create a situation in which health is continuously deprioritised, delayed, or made difficult to act on, despite awareness and intention.

### 1. When caring for others becomes a barrier to one’s own health

In this theme, participants described how their concern for others, such as children, elderly parents, or family members living abroad, shaped their everyday practices and often limited their possibilities to prioritise their own well-being. Worrying for others often led to caregiving responsibilities and emotional burdens, and was described as a constant presence that shaped daily health-related decisions and behaviours. Rather than existing in isolation from daily health behaviours, these responsibilities functioned as significant barriers, draining individuals’ energy, disrupting routines, and reducing their readiness to engage in health-promoting activities. Across participants’ accounts, caring for others was described as both a moral obligation embedded in cultural expectations that produced meaning and as a set of demands that were often prioritised over personal well-being.

#### Transnational worries

Transnational worries related to political instability and hardship experienced by relatives in their countries of origin created an ongoing emotional strain that became physically embodied. Participant 3 described witnessing how, despite stable lives in Sweden, both he and his friends experienced deteriorating health due to worry for relatives abroad, including developing hypertension and heart problems: “It also makes you feel unwell… when I start thinking, get stressed, my blood pressure goes up”. Other participants experienced how emotional overwhelm was part of shaping unhealthy habits. For example, participant 5 explained: “I feel sad… Then you start eating late. It becomes a routine… that’s why I am overweight”.

For many, extensive transnational concerns, along with caregiving responsibilities, create competing priorities that leave little time or energy for themselves. Participant 8 described “You have to eat, wash [clothes], prepare, fix things. You might also have another family far away [abroad] who is also your priority”. Such responsibilities expanded caregiving from the immediate household in Sweden to their home countries, increasing participants’ emotional burden. Participant 2 emphasised the burden of caring for others: “I can’t think only about myself; I have to think about everyone else [abroad and here]”.

#### The paradox of caring for others

Caring responsibilities within the household also placed significant demands on the participants, yet were described as meaningful and closely tied to satisfaction. On the one hand, many participants described juggling childcare, household duties, work, and studies, leaving little time for themselves. For instance, participant 6 spoke of the impossibility of engaging in physical activity beyond home-based routines because her days were structured entirely around caring for her children: “I can’t… I can’t go to the gym because I have two children”. On the other hand, participants described caring for others as a natural responsibility and a source of joy that positively impacts their health and well-being. “If I have time at home, I sit with the children. We do fun things with the children. I think that’s good too for health and for your thoughts” (participant 7).

The paradox was grounded in how caregiving was described ambivalently. Some noted how personal and culturally significant it was to be responsible for older or ill relatives, and others described the emotional and practical burdens of these responsibilities, as well as the juggling of them alongside other expectations. Participant 3 described the cultural embeddedness of caring for others:

“I do not have those kinds of barriers or anyone preventing me, saying that I am not allowed to do things. Rather, it is something within myself, or perhaps, as you can see, it is cultural. I feel that I should also be there. Yes, because I think the children or the family might feel I am not paying enough attention to them. So, it is really myself who feels responsible. These are really my own thoughts, but perhaps also related to my cultural background”.

Participant 1 described the emotional toll of caring for a seriously ill mother: “The mental stress is much harder… this has made me put myself very much on pause”. Participant 8, who was both a primary caregiver for his mother and the eldest sibling, described feeling unable to live his own life: “I feel like I’m the only one taking care of my mother… Then you can feel: my God, how am I going to cope?”.

Collectively, participants described how caring for others limited their engagement in self-care activities aimed at maintaining health and well-being. Caring for others was also meaningful and enjoyable, yet potentially burdensome due to multiple responsibilities rooted in cultural expectations and transnational ties. These responsibilities serve as both barriers and enablers in participants’ lives, reducing their energy while providing meaning and shaping their everyday possibilities for engaging in health-promoting activities.

### 2. When economic conditions hinder engagement in health-promoting behaviours

Participants described how, despite positive prior experiences with regular exercise and an understanding of the benefits of health-promoting behaviours, both financial and psychological barriers limited their ability to engage in such activities. Economic constraints limited access to certain forms of physical activity, while ongoing stress undermined their motivation to initiate or maintain healthy routines. Participants expressed a desire to engage in exercise or healthy eating, yet financial insecurity and emotional strain consistently undermined these intentions.

#### Stress undermines motivation

Participants described the overall demands/burdens of everyday life (i.e., practical/work or studies, emotional, and private) as a powerful force that led to stressful everyday life. These demands were perceived as inducing both stressful situations and emotional stress and disrupting participants’ motivation and efforts to sustain new routines, even when participants began health-promoting efforts with strong intentions. Overall, the high demands of everyday life and the consequences they brought were described as barriers that pushed participants back into familiar but less healthy routines. Participant 10 noted: “Motivation is strong when I start. But then stress affects me a lot… and then you kind of let go of the diet and start eating again, like things you maybe shouldn’t eat”. Others described difficulties in sustaining health-related behavior over time: “I have tried different ways of eating and exercising, but then I tend to lose it” (participant 1).

Participants also spoke about consciously slowing down or postponing engagement in health-promoting activities when overall practical and emotional burdens in everyday life became overwhelming, as an attempt to take care of themselves. Participant 14 shared her strategy of self-regulation: “I think about myself and that stress isn’t good… then I say: no, deep breaths, sit down, do it tomorrow. I try to slow myself down”. While this strategy reflects a conscious way to reduce emotional stress and manage everyday life burdens, it also illustrates how stressful situations and emotional stress not only drain motivation but also can delay engagement in health-supporting behaviours.

#### Economic constraints limiting access

Across participants’ responses, financial insecurity further limited their opportunities to engage in structured forms of physical activity. Even those who valued exercise and felt its benefits could not always afford certain activities, such as swimming at the municipal pool or at the local gym. Participant 1 explained: “I know how good I feel from it [physical activity], but I don’t have the finances for things that cost a lot of money”. Another participant explained how financial insecurity negatively affected her daily functioning: “I want planning for it [health-promoting activity], but it [the money] isn’t enough […] Finances affect your health when you sleep. Sometimes it also gets into your head. So you sleep poorly, you eat poorly, and your mood. it’s bad; everything is affected” (participant 4).

Although walking was described as cost-free and accessible, and as beneficial for their physical and psychological well-being, competing demands still diminished participants’ ability to enjoy this activity. Participant 6 noted: “I walk to school for my health so that I feel better. It’s just that I have a lot of stress. yes, a lot of stress at school, with my studies, and I’m busy, and there’s a lot of stress there”. Another described how walking improved her well-being but was often displaced by a lack of time, which resulted in stress: “On the days I go for a walk, I feel better. But on the days you don’t have time, that’s when you feel more stressed and depressed” (participant 3).

#### Social support as a partial buffer

At the same time, participants emphasised the important role of social support in facilitating participation in health-promoting activities. Walking with others, particularly those who offered encouragement or shared similar health goals, helped counteract internal barriers such as low motivation, feelings of stress, or discomfort with being outdoors. Participant 15 appreciated how her neighbour supported her: “We go together […]. Sometimes she forces me; sometimes I come along. I don’t go on my own. I don’t like the outdoors”. Another described a friend who regularly invited her to join walks: “I have a friend who usually says, ‘come and join me.’ I’ve done it sometimes and sometimes not, but my friend would really like me to come along and be more active” (participant 17). However, social encouragement was not always enough to overcome participants’ everyday pressures and prioritisation under constrained circumstances. As participant 3 explained, despite frequent invitations to swim or exercise together: “But unfortunately, I’m usually the one who backs out. It doesn’t turn out the way you plan. Life is tough, and I work while also having a family, so it becomes a bit difficult”.

Taken together, participants described sustained efforts to engage in health-promoting behaviours, but these efforts were consistently undermined by financial constraints, competing demands, and everyday burdens, leading to stressful everyday situations and emotional stress. While social support offered some encouragement, it was often not enough to offset the cumulative everyday demands of even cost-free activities such as walking.

### 3. When health-promoting spaces become stressful and unsafe

Participants described how environments typically framed as health-promoting, such as local gyms, walking paths, and neighbourhoods, were instead experienced as stressful, uncomfortable, or unsafe. These environmental, sensory, and social stressors significantly reduced participants’ willingness and ability to engage in health-promoting activities, even when they were motivated or aware of the associated health benefits.

#### Overwhelming health-promoting spaces

Participants explained that gyms, despite being conventional sites for physical activity, evoked feelings of stress, discomfort, or sensory overload. Participant 3 described feeling overwhelmed by the crowded, fast-paced, and socially charged atmosphere:

I have had a gym membership for almost a year, but I have only been there about three times, so I am not very engaged. I just cannot manage it. I find the environment there very stressful because there are so many people, some are running, and others are doing different things, and most people say it is very engaging. That creates stress for me.

Another participant highlighted that despite having a gym with a room only for women, which promotes safety, the gym’s sensory environment became a barrier to engaging in health-promoting activities:

I do not like going to the gym. I am very sensitive to smells. I suffer from migraines, and certain perfumes or, you know, different scents affect me, but I am also sensitive if people are not clean. Where I lived before, we had a gym with a separate room for women, but women do not always smell good either, and there can be even more perfume (participant 18).

Others expressed a need for spaces where they could feel safe and comfortable rather than judged or pressured. As participant 4 explained: “The most important thing is that you feel safe when you go there”. These descriptions highlight that beyond economic accessibility, sensory and emotional safety are essential conditions for participation.

#### Neighbourhood unsafety

In addition to indoor environments, participants described how neighbourhood safety concerns limited their ability to walk or be physically active outdoors. Experiences of crime, visible insecurity, or fear for children’s safety shaped their possibilities to engage in health-promoting activities. Participant 9 shared ongoing worry triggered by environmental cues: “You keep thinking all the time, like, have they ended up somewhere? You become scared like that. As soon as you hear a helicopter or police cars, you wonder, what is happening?”.

Others described how news about local violence has started to affect their engagement in activities such as walking, as one participant describes: “It may not be very wise to walk alone. I used to do that quite a lot. I would walk back and forth after work. But now I have started to rethink it after what has happened, like if you might run into someone criminal?” (participant 12).

Participants described this growing sense of insecurity in their local neighbourhoods: “You feel worried all the time and have to check what is going to happen, who it is or something” (participant 4); “During the daytime I feel that it is safe, but in the evening I find it a bit uncomfortable” (participant 10). Parents, in particular, felt concern due to increasing media reports of gang violence in their neighbourhoods, which can also restrict children’s participation in diverse activities.

Unfortunately, issues with gangs and shootings exist across all of Sweden. And that makes things feel more worrying, especially when my son goes out. You become worried that something might happen, and you want to keep track of where he is, who he is with, when he will come home, and so on (participant 17).

Overall, participants’ descriptions show how both indoor and outdoor environments can shape their sense of comfort and safety to engage in health-promoting activities. These spaces can be experienced as stressful or overstimulating, becoming emotionally charged and amplifying existing challenges, and in some cases cumulatively limiting opportunities to be active across multiple settings (e.g., at home, in the neighbourhood, or at the gym).

## Discussion

The aim of this study was to explore how residents at high risk of stroke experience and engage in everyday health-promoting activities within their neighbourhoods. Previous research has demonstrated that socioeconomic inequalities are deeply embedded in stroke risk and outcomes [1, 3], and that social gradients in modifiable risk factors are patterned by socioeconomic conditions [6]. While such research has established what drives inequalities, the present findings contribute by illustrating how these inequalities are navigated, negotiated, and reproduced in everyday life. In doing so, the study responds to calls for more research exploring the lived experiences of people in disadvantaged contexts across the stroke pathway [21, 22]. These findings complement epidemiological studies from Sweden that demonstrate persistent inequalities in stroke incidence and outcomes [4, 5] by showing how these inequalities are reproduced through everyday practices (i.e., responsibilities, expectations, and priorities) shaped by competing demands.

Across the findings, engagement in health-promoting practices emerges as shaped by personal, interpersonal, and contextual conditions that define what is possible and expected in participants’ contexts. Health is therefore not enacted as an individual responsibility alone, but as something negotiated within family responsibilities, emotional burdens, and practical circumstances. This challenges individualistic models of health behaviour and aligns with social determinants of health perspectives that emphasise how structural and relational conditions shape health opportunities [38, 39].

### When caring for others becomes a barrier to one’s own health

A central contribution of this study is the identification of caregiving as complex, encompassing both meaningful and constraining forces. Participants described caregiving responsibilities as morally grounded, culturally embedded, and closely tied to identity and purpose. This aligns with broader research showing that caregiving is both a source of fulfilment and a strain [40]. At the same time, these responsibilities often limit time, money, energy, and opportunities to engage in health-promoting activities. Health promotion research has identified family responsibilities and competing roles as key barriers to physical activity, particularly among disadvantaged populations [41, 42]. Importantly, the findings extend the existing literature by foregrounding care responsibilities that extend beyond one’s own home, including extended family and relations in other countries.

Participants described ongoing emotional strain related to family members abroad, which became embodied in stress-related symptoms and shaped everyday routines. This resonates with emerging research on transnational families, which demonstrates how obligations across borders produce sustained emotional and financial pressures [43]. Caregiving can thus be understood as a dominant and meaningful activity that structures daily life and often displaces opportunities for restorative or health-promoting activities [44]. These findings further highlight the ambivalence of caregiving: while meaningful and health-supporting in some respects, it simultaneously constrains self-care. This ambivalence underscores the need to move beyond binary understandings of caregiving as either a burden or a benefit and instead recognise it as a relational and context-dependent practice shaped by cultural expectations and structural conditions.

### When economic conditions hinder engagement in health-promoting behaviours

Participants’ accounts demonstrate that engagement in health-promoting activities is not limited by a lack of prior experience, knowledge, or motivation but by cumulative triggers of stress and economic precarity. While participants expressed awareness of health risks and intentions to engage in healthy behaviours, these were repeatedly disrupted by competing demands. This aligns with research indicating that individuals with lower SES encounter structural barriers that hinder their ability to act on health knowledge [25]. It may also explain why prevention efforts often do not effectively reach disadvantaged groups [19, 20], especially when they focus solely on individual behaviour or health literacy.

Feelings of stress emerged as a central mechanism through which these constraints operate. Rather than being an individual psychological factor, stress arose from the intersection of caregiving responsibilities, financial insecurity, and everyday demands. This aligns with literature emphasising how chronic stress undermines sustained health behaviour change [45–47]. Likewise, economic constraints restricted access to structured physical activities such as gyms or swimming, reinforcing existing inequalities in health opportunities. Notably, even cost-free activities such as walking were constrained by time scarcity, fatigue, and competing priorities. This suggests that accessibility needs to be understood beyond financial cost alone, encompassing time, energy, and cognitive resources. Taken together, these cumulative constraints limit opportunities for health promotion from both a physical perspective (e.g., increased stroke risk) and a mental health perspective, given the established preventive role of physical activity in mitigating mental ill-health [48, 49].

While social support was described as a facilitator, its role was often limited. Encouragement from others did not consistently translate into sustained engagement, particularly under conditions of high cumulative burden. This aligns with research suggesting that social support operates through complex pathways and may not directly lead to behaviour change [7]. Together, these findings point to the limits of interventions that rely primarily on social encouragement without addressing broader structural constraints.

### When health-promoting spaces become stressful and unsafe

A key contribution of this study lies in illustrating how health-promoting spaces are experienced. Participants’ accounts challenge the assumption that environments such as gyms or neighbourhood walkways are inherently supportive of health. Instead, these spaces were often described as stressful, overwhelming, or unsafe. Gyms, for example, were experienced as socio-material environments shaped by sensory overload, social norms, and expectations of performance. This aligns with qualitative research indicating that physical activity settings can lead to discomfort, exclusion, or a lack of belonging, particularly among disadvantaged groups [50]. These findings suggest that positioning the gym as a primary site for health promotion risks overlooking the diversity of lived experiences and highlights the need for more inclusive and varied health-promoting activity options.

Similarly, neighbourhood environments were shaped by perceptions of safety and insecurity. Participants described how fear of crime and exposure to media reports influenced their willingness to engage in outdoor activities such as walking. This is consistent with research demonstrating that perceived safety is a critical determinant of physical activity, particularly in low-income areas [51, 52].

Together, these findings suggest that health-promoting spaces are not neutral but are experienced differently depending on prior experiences and contextual conditions. What is commonly framed as a resource for health promotion may simultaneously be perceived as alienating or unsafe, thereby contributing to a cumulative reduction in opportunities for being active across environments and reinforcing inequalities in engagement in meaningful health-promoting activities. Furthermore, the findings indicate that engaging in such activities can become an additional burden when time constraints, financial limitations, fatigue, and caregiving responsibilities constrain the conditions necessary for enjoyment, meaning, and social connection, which are critical for sustained engagement [43].

### Strengths and limitations

A key strength of this study is the inclusion of a relatively large number of participants, given that this population is often underrepresented in research. Recruiting individuals residing in low SES urban neighbourhoods and at high risk of stroke was methodologically challenging, yet the use of tailored, context-sensitive recruitment strategies was central to participation. This strengthened the study by generating rich, contextually grounded insights. The use of reflexive thematic analysis further allowed for an in-depth exploration of the multi-level factors shaping health practices, supporting theoretically informed interpretation.

However, several challenges should be acknowledged. The analysis involved translation of quotes from Swedish into English, which may have resulted in subtle losses of meaning, particularly for culturally embedded expressions [53]. While this was mitigated through collaboration among researchers with strong proficiency in Swedish, translation remains a process that can limit the expression of situated expressions and nuances. Furthermore, variation in participants’ language proficiency may have influenced both what was expressed and how it was communicated, potentially privileging those more comfortable in the interview setting and language. Yet several strategies were used to provide more time for those needing it to articulate their ideas [54]. Finally, while the study provides in-depth insights into everyday experiences, it does not fully account for how intersecting factors such as gender, age, and migration background shape these experiences, suggesting a need for further intersectional research.

## Conclusion

This study demonstrates that everyday efforts to stay healthy among individuals at high risk of stroke in low SES urban contexts are shaped by interconnected personal, social, and contextual constraints. Rather than reflecting a lack of motivation or awareness, participants’ experiences reveal ongoing negotiations of caregiving responsibilities, financial pressures, and environments that are not consistently supportive of health-promoting practices. As such, addressing inequalities in stroke requires moving beyond individual behaviour change toward context-sensitive, multi-level approaches that acknowledge and respond to the conditions shaping everyday life. Health promotion strategies should be designed to be flexible, meaningful, integrated into daily routines, and supported by family and work/study settings. Ultimately, without structural and contextually responsive approaches, those at highest risk of stroke are likely to remain least able to engage in health-promoting efforts. Advancing health equity, therefore, depends on creating conditions in which health-promoting practices are not an added burden, but a realistic, engaging, and supported part of everyday life.

## Data Availability

No datasets were generated or analysed during the current study.
